# Non-infectious Hepatic Cystic Lesions: A Narrative Review

**DOI:** 10.2174/0115734056358195250714053120

**Published:** 2025-09-17

**Authors:** Adem Ceri, Andreas Busse-Coté, Delphine Weil, Eric Delabrousse, Vincent Di Martino, Paul Calame

**Affiliations:** 1 Department of Radiology, University of Bourgogne Franche-Comté, CHU Besançon, 25030 Besançon, France; 2 Department of Hepatology, University of Bourgogne Franche-Comté, CHU Besançon, 25030 Besançon, France; 3 EA 4662 Nanomedicine Lab, Imagery and Therapeutics, University of Franche-Comté, Besançon, France

**Keywords:** Hepatic cyst, Complicated cyst, Mucinous cystic neoplasm, Polycystic liver disease, Hamartoma, Caroli disease, Ciliated hepatic foregut cyst

## Abstract

Hepatic cysts are commonly encountered in clinical practice, presenting a wide spectrum of lesions that vary in terms of pathogenesis, clinical presentation, imaging characteristics, and potential severity. While benign hepatic cysts are the most prevalent, other cystic lesions, which can sometimes mimic simple cysts, may be malignant and pose significant clinical challenges. Simple biliary cysts, the most common type, are typically diagnosed using ultrasound. However, for complex lesions, advanced imaging modalities such as Computed Tomography (CT) and Magnetic Resonance Imaging (MRI) are crucial. In ambiguous cases, additional diagnostic tools such as contrast-enhanced ultrasound (CEUS), Positron Emission Tomography (PET), cyst fluid aspiration, or biopsy may be necessary. Understanding the nuances of these cystic lesions is crucial for accurate diagnosis and management, as it distinguishes between benign and potentially life-threatening conditions and informs the decision on appropriate treatment strategies. Non-parasitic cysts encompass a range of conditions, including simple biliary cysts, hamartomas, Caroli disease, polycystic liver disease, mucinous cystic neoplasms, intraductal papillary mucinous neoplasms, ciliated hepatic foregut cysts, and peribiliary cysts. Each type has specific clinical and imaging features that guide non-invasive diagnosis. Treatment approaches vary, with conservative management for asymptomatic lesions and more invasive techniques, such as surgery or percutaneous interventions, reserved for symptomatic cases or those with complications. This review focuses on non-parasitic cystic lesions, exploring their pathophysiology, epidemiology, risk of malignant transformation, treatment options, and key findings from imaging diagnosis.

## 1 INTRODUCTION

Hepatic cysts are frequently encountered in daily clinical practice and encompass a diverse group of lesions that vary based on pathogenesis, clinical presentation, imaging characteristics, and potential severity. Benign hepatic cysts are the most common; however, other lesions-sometimes challenging to distinguish from simple cysts-can be malignant and even life-threatening. The most widely used classification system categorizes hepatic cysts into infectious and non-infectious origins, further dividing them into parasitic and non-parasitic types. Pyogenic abscesses are often excluded from diagnostic algorithms due to their distinct clinical and morphological features. This review focuses exclusively on non-parasitic cystic lesions. For each lesion type, we discuss epidemiological data, natural history (including the risk of malignant transformation), imaging findings, and treatment options.

Abdominal ultrasound typically suffices for diagnosing most simple biliary cysts. However, other lesions necessitate advanced medical imaging techniques, such as Computed Tomography (CT) or Magnetic Resonance Imaging (MRI). For ambiguous diagnostic scenarios or to guide therapeutic decisions, additional specialized tools-including contrast-enhanced ultrasound (CEUS), Positron Emission Tomography (PET), cyst fluid aspiration, or biopsy-may prove beneficial and warrant consideration.

## 2 BILIARY CYSTS

Simple biliary cysts are currently the most common type of hepatic cystic formations and can be located anywhere within the liver. The term “biliary cyst” is commonly used due to the presence of an epithelium similar to that of the bile ducts [1]. However, this term is considered somewhat inappropriate since these cysts do not communicate with the biliary tract [2]. The alternative term “simple cyst,” introduced in the 1980s to differentiate them from true cystic dilations of the bile ducts seen in Caroli syndrome and congenital hepatic fibrosis, is less commonly used [3].

### 2.1 Natural History

The exact origin of biliary cysts is not fully understood, but they are likely congenital, possibly resulting from cystic dilation of aberrant bile ducts or microhamartomas. Simple liver cysts were once considered rare but are now found in 2-5% of ultrasound studies and up to 18% of CT scans due to the sensitivity of detection [4].

These cysts are rare before the age of 10, increase with age (peaking between 50-60 years old), and are more common in women [4]. They are often small but can grow very large and become symptomatic. Enlargement occurs in less than 20% of cases, with growth factors still poorly understood, though hormonal influences are suspected due to the female predominance.

### 2.2 Imaging

Most simple hepatic cysts are asymptomatic and incidentally discovered. On ultrasound (US), they typically appear as anechoic, round lesions with well-defined borders, no visible wall, and posterior acoustic enhancement. Critical negative diagnostic signs include the absence of internal septations, intracystic structures, and calcifications, although these features can occasionally be present and misleading (Table **1**). Outside of these situations, complications, or specific contexts, Ultrasound is generally the only necessary and sufficient imaging modality for diagnosis [5].

**Fig. (1):**
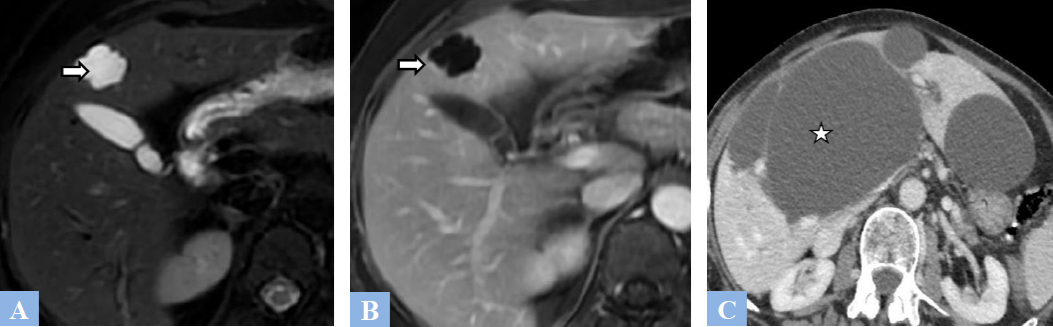
Examples of simple biliary cysts. **A.** T2-weighted fat-suppressed MR image in the axial plane showing a biliary cyst appears as a T2 hyperintense lesion with lobulated contour (arrow). **B.** T1-weighted fat-suppressed MR image in the axial plane after gadolinium administration at the portal phase of enhancement. No wall enhancement observed (arrow). **C.** CT image in the axial plane at portal phase of enhancement showing well-defined hypodense cystic lesions (asterisk) without walls, located in segment IV and the left lobe.

On CT, biliary cysts appear as rounded, hypodense lesions without density changes (20 HU) after contrast administration (Fig. **1**). In cases of significant hepatic steatosis, distinguishing cysts may be challenging [6].

On MRI, biliary cysts are homogeneous, hypointense on T1, and strongly hyperintense on T2, consistent with fluid-filled lesions [7] (Fig. **1**).

### 2.3 Complications

**Fig. (2):**
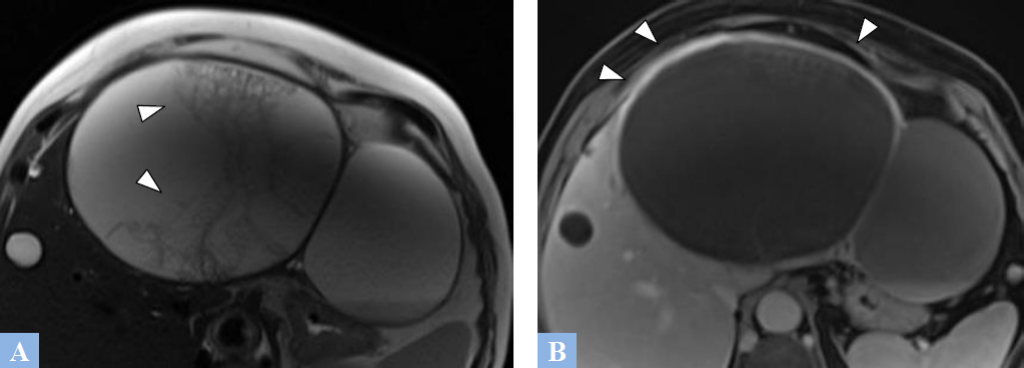
Infected biliary cyst in a 63-year-old female patient presenting with febrile pain in the right hypochondrium. MRI features. **A.** T2-weighted MR image in the axial plane. Cystic lesion in segment IV with a thick wall and heterogeneous content (arrowhead). **B.** T1-weighted fat-suppressed MR Image in the axial plane after gadolinium administration at the portal phase of enhancement. Contrast enhancement of the cyst wall is consistent with cyst infection (arrowheads).

**Fig. (3):**
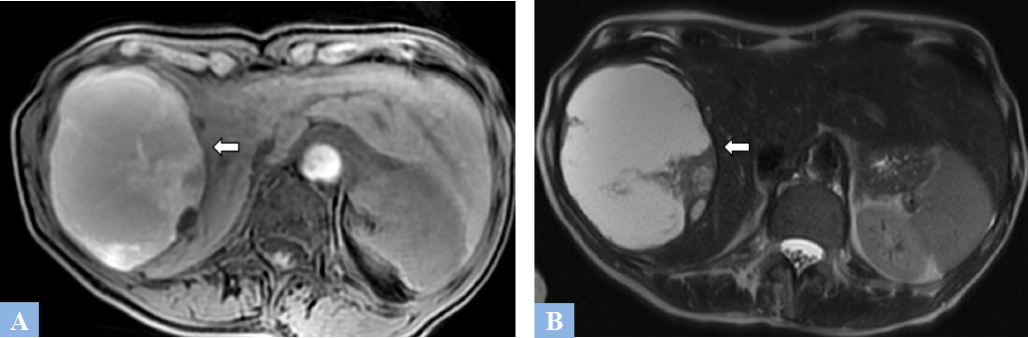
Hemorrhagic biliary cyst in a 76-year-old female patient presenting with right hypochondrium pain. MRI features. **A.** T1-weighted fat-suppressed MR image in the axial plane. Cystic lesion in the right liver with spontaneous T1 hyperintensity, consistent with hemorrhage. **B.** T2-weighted fat-suppressed image in the axial plane showing heterogeneous content of the hepatic cyst, consistent with hemorrhage.

Complications are rare and predominantly occur in large cysts [6]. The following complications are listed in decreasing order of frequency:

- **Pain**: Large cysts stretching Glisson's capsule may cause discomfort. Ultrasound-guided drainage can confirm the cyst as the source of pain if it provides relief.
- **Organ Compression:** This can affect digestive (gastroduodenal), vascular (causing inferior vena cava syndrome or portal hypertension), renal, or biliary structures. Compression of the biliary convergence by a large central hepatic cyst, potentially leading to reversible cholestatic jaundice following cyst drainage, is the most common form of mechanical compression.
- **Intracystic Hemorrhage (Fig. **2**):** Presents with right upper quadrant pain and cyst enlargement. Ultrasound may reveal heterogeneous and mobile cyst contents, and MRI shows hyperintense T1 and heterogeneous T2 signals indicative of hemorrhage. Differentiating between hemorrhagic biliary cysts and cystadenomas can be challenging [8].
- **Bacterial Infection (Fig. **3**):** May be iatrogenic or spontaneous, presenting similarly to hepatic abscesses. When involving an enterobacterium, a digestive entry point should be investigated.
- **Wall Ruptures**: These are rare and sudden, occurring into the peritoneal cavity, bile ducts, or duodenum, often triggered by intracystic hemorrhage [9]. An intraperitoneal rupture in a woman on estrogen-progestin contraception can be misdiagnosed as a ruptured hemorrhagic hepatic adenoma.

### 2.4 Treatment

Uncomplicated biliary cysts do not require treatment or follow-up [2]. Aspiration alone is primarily diagnostic, but it is essential in infected cysts, either alone or in conjunction with drainage. For symptomatic cysts, the primary treatments are percutaneous sclerotherapy, which involves fluid aspiration followed by the injection of a sclerosing agent [10], and surgical intervention, typically performed *via* laparoscopic fenestration. Sclerotherapy aims to halt secretion by destroying the cyst epithelium, although it carries risks of pain and recurrence. Surgical fenestration involves removing the protruding part of the cyst, while total cystectomy is reserved for cases with diagnostic uncertainty or complications [11]. A systematic review indicated that sclerotherapy and surgery have similar rates of symptom persistence, and surgery is recommended mainly when percutaneous treatment fails; however, further randomized studies are needed to confirm these recommendations [12].

## 3 HAMARTOMAS

Hamartomas, also known as Von Meyenburg complexes, biliary hamartomas, or biliary microhamartomas, are small, non-encapsulated nodules (typically < 5 mm) that are well-circumscribed. These lesions are thought to arise from incomplete remodeling of the ductal plate during the late stages of liver development when small intrahepatic bile ducts form [13], and are usually located near portal areas.

Biliary hamartomas are the most common form of fibropolycystic liver disease, affecting about 0.6% to 2.8% of the general population. They are associated with other conditions such as simple biliary cysts, polycystic kidney disease, congenital hepatic fibrosis, and Caroli disease. Clinically, biliary hamartomas are benign and remain asymptomatic throughout life, with no need for treatment or histological confirmation in patients with typical imaging findings.

### 3.1 Imaging

Hamartomas are often incidentally discovered during imaging for unrelated reasons. They usually appear as small, cystic lesions (less than 1 cm) that are well-defined, relatively uniform in size, and scattered throughout the hepatic parenchyma. When small cystic lesions occur in large numbers, this condition is referred to as hamartomatosis. On ultrasound, small hamartomas generally appear echogenic, and millimetric lesions may be misinterpreted as diffuse heterogeneous hepatic echotexture. Larger hamartomas may appear hypoechoic or anechoic, sometimes with a “comet tail” artifact, which can be mistaken for intrahepatic stones [14, 15]. On CT, Von Meyenburg complexes appear as multiple hypodense lesions that do not enhance after contrast administration [16]. MRI Fig. (**4**) shows well-defined, hypointense lesions on T1-weighted images and distinctly hyperintense on T2-weighted images, creating a characteristic “starry sky” or “snowstorm” appearance on T2 [17, 18]. The bile ducts, both intra- and extrahepatic, are typically not dilated and do not communicate with the cysts [6].

Characterizing hamartomas can be challenging due to their small size, and distinguishing them from small hepatic metastases often requires cross-sectional imaging, as ultrasound alone is insufficient [19]. Key diagnostic features include the uniform size of the cysts and the absence of contrast enhancement in the lesions. However, partial enhancement of the cyst wall post-contrast has been described, depending on the pericystic stroma and the presence of abnormal portal branches. In oncological contexts, a biopsy may be considered, although it often yields limited results due to the small lesion size, and ongoing morphological follow-up is essential [20].

### 3.2 Natural History

Complications of hamartomas are extremely rare, with only one reported case of liver transplantation for recurrent cholangitis possibly misattributed to hamartomas [21]. While hamartomas are generally benign, case reports suggest a potential risk of transformation into cholangiocarcinoma. Histological observations have revealed transitions from benign to hyperplastic, dysplastic, and invasive carcinoma within resected specimens, supported by molecular analyses that demonstrate shared mutations between cholangiocarcinoma and hamartomas [22-24]. However, specific genetic changes driving this transformation remain unclear, as BRAF mutations found in some cholangiocarcinomas are not present in Von Meyenburg complexes [25].

## 4 CAROLI DISEASE

Caroli disease is a benign congenital disorder characterized by segmental cystic dilation of the intrahepatic bile ducts, affecting one or both liver lobes. First described in 1958 by Jacques Caroli, its incidence is estimated at 1 in 1,000,000. The disease affects both males and females equally, with over 80% of cases diagnosed before the age of 30.

There are two forms of Caroli disease: the more common form, associated with congenital hepatic fibrosis and known as Caroli syndrome, and a simpler form that occurs independently. The isolated form is less common and typically involves more localized areas of the liver. Caroli disease is associated with an increased incidence of bile duct stones, recurrent cholangitis, and liver abscesses [26]. Cirrhosis is typically absent at diagnosis, although portal hypertension secondary to congenital hepatic fibrosis may already be present. The disease is also associated with various renal conditions, including autosomal recessive polycystic kidney disease in children, medullary sponge kidney, and Cacchi-Ricci disease [3].

### 4.1 Origin and Natural History

Caroli disease is a genetic disorder primarily inherited in an autosomal recessive pattern. Several theories have been proposed to explain the development of cystic dilations of the bile ducts, including neonatal occlusion of the hepatic artery leading to biliary ischemia, abnormal development of the biliary epithelium and surrounding tissues, and incomplete involution of the ductal plate surrounding the portal branches during embryonic development [8]. Among these, the incomplete ductal plate involution hypothesis is most consistent with the observed pathology.

Caroli disease significantly increases the risk of cholangiocarcinoma, with a 100-fold higher incidence than the general population, ranging from 5% to 10%. Although present from birth, the disease can remain asymptomatic for many years or even throughout life; however, symptoms can also emerge as early as childhood. When symptomatic, patients often experience a marked decline in quality of life, with common symptoms including recurrent fever, jaundice, and right upper quadrant pain. Acute cholangitis is the most frequent clinical presentation (64% of patients) and is the leading cause of mortality, often caused by Gram-negative bacilli that become resistant to antibiotics over time. Portal hypertension due to congenital hepatic fibrosis occurs in 20% to 50% of cases, potentially leading to hypersplenism and esophageal variceal rupture [26].

Biochemically, chronic cholestasis is commonly observed, with liver function generally preserved for an extended period.

### 4.2 Imaging

The key to the radiological diagnosis of Caroli disease is demonstrating a connection between cystic lesions and normal bile ducts [6]. While this can be visualized on ultrasound or CT, MR cholangiography is the most effective method as it provides a comprehensive view of the entire biliary tree.

**Fig. (5):**
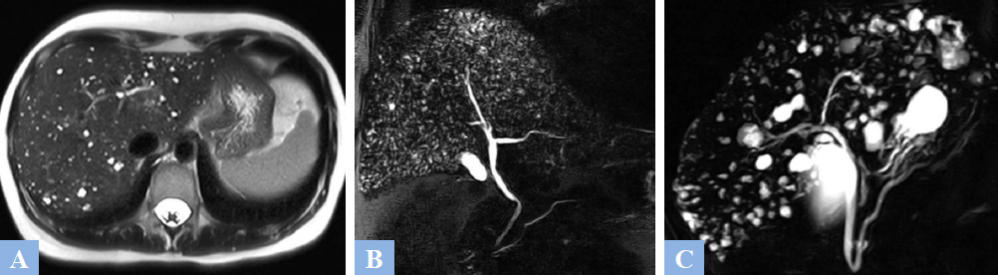
Caroli syndrome in a 47-year-old patient was discovered during an episode of cholangitis. **A.** T2-weighted MR image in the axial plane showing hepatic cystic lesions with T2 fluid hyperintensity, communicating with the bile ducts. **B.** 2D radial MR cholangiography sequences. Cystic dilations of the bile ducts (arrowhead) without stenosis at the origin of the dilations. **C.** T1-weighted fat-suppressed MR Image in the axial plane after gadolinium administration at the portal phase of enhancement. “Dot sign” (red circle) corresponding to the visualization of a portal vein within a hepatic cyst.

MR cholangiography typically reveals three forms of Caroli disease: 1) multiple cystic ectasies combined with fusiform dilations, 2) isolated fusiform dilations with multiple stones, and 3) a localized form in the left lobe with combined dilations, cysts, and multiple stones [8]. On contrast-enhanced CT scans, a specific “dot sign” indicative of a small portal branch surrounded by dilated bile ducts can be observed [27] (Fig. **5**). This sign is also identifiable on MRI [28] and Doppler ultrasound. In cases associated with congenital hepatic fibrosis, the liver may appear dysmorphic, with additional morphological signs of portal hypertension, including venous shunts.

The overlapping features with cholangiocarcinoma make its diagnosis challenging in Caroli disease patients, highlighting the need for regular imaging follow-up [29]. Comparing sequential MR cholangiographies can help detect mass effects or distortions in the dilated bile ducts, aiding early detection of malignancy.

### 4.3 Treatment

The treatment of Caroli disease depends on the clinical manifestations and the extent of biliary involvement. Conservative management is preferred, involving the use of antibiotics for cholangitis episodes, ursodeoxycholic acid, and endoscopic removal of accessible intrahepatic stones. Surgery, such as right or left hepatectomy, is reserved for symptomatic cases that do not respond to conservative treatments, with low morbidity and negligible mortality [30].

For diffuse bilateral involvement with recurrent cholangitis, abscesses, or secondary biliary cirrhosis, liver transplantation is considered, offering good long-term outcomes and preventing cholangiocarcinoma, with no recurrence in the graft [31]. Transplantation is contraindicated in the presence of cholangiocarcinoma due to high recurrence rates. For cases involving congenital hepatic fibrosis and severe portal hypertension unmanageable by Transjugular Intrahepatic Portosystemic Shunt (TIPS) [32], transplantation may also be indicated.

## 5 POLYCYSTIC LIVER DISEASE

Polycystic Liver Disease (PLD) is a rare hereditary disorder characterized by the presence of multiple diffuse cysts within the liver. It may occur in isolation (isolated PLD) or, more commonly, in association with Autosomal Dominant Polycystic Kidney Disease (ADPKD) or the less frequent Autosomal Recessive Polycystic Kidney Disease (ARPKD). The precise mechanisms of cyst formation in PLD are not fully understood. In ADPKD, cystogenesis involves dysfunction of the primary cilia in biliary epithelial cells, which normally regulate intracellular calcium levels. A reduction in intracellular calcium activates several pathways that promote cholangiocyte proliferation and cyst fluid secretion, placing polycystic diseases within the category of ciliopathies [33]. However, the congenital bile duct dysplasia observed in PLD suggests the involvement of additional, yet unidentified mechanisms. Consequently, PLD is often classified among congenital cystic diseases of the intrahepatic bile ducts, alongside hamartomas and Caroli disease [34].

### 5.1 Epidemiology and Genetics

#### 5.1.1 PLD Associated with ADPKD

Autosomal dominant polycystic kidney disease is a common genetic disorder with a prevalence of 1 in 1,000, and Polycystic Liver Disease (PLD) is its most common extra-renal manifestation. ADPKD is caused by mutations in the PKD1 and PKD2 genes, leading to disrupted intracellular calcium regulation and the activation of pathways that promote cyst formation [35].

#### 5.1.2 PLD Associated with ARPKD

Autosomal recessive polycystic kidney disease is rare, with an incidence of 1 in 20,000, often affecting children and associated with severe complications. It is primarily linked to mutations in the PKHD1 gene, which encodes fibrocystin [36].

#### 5.1.3 Isolated PLD or Autosomal Dominant Polycystic Liver Disease

Isolated PLD is an autosomal dominant condition with low penetrance, involving various genes related to protein glycosylation, folding, and quality control, such as PRKCSH, SEC63, and others. Genetic causes remain unidentified in many cases, and recent research suggests that cholangiocyte autophagy may play a role in cyst formation, presenting a potential therapeutic target [36, 37].

### 5.2 Clinical Manifestations

In Polycystic Liver Disease (PLD) or hepatorenal polycystic disease, hepatic cysts are uncommon before the age of 20 but are present in over 80% of individuals after the age of 60. Liver volume increases by approximately 2% every 6 to 12 months, but most patients remain asymptomatic regardless of the type of PLD [36]. About 20% of patients develop symptoms, which may include dyspnea due to thoracic displacement or compression of the right heart chambers, edema from inferior vena cava compression, early satiety, abdominal distension, gastroesophageal reflux, malnutrition with weight loss and sarcopenia due to chronic gastric compression, and back or right upper quadrant pain from hepatomegaly affecting adjacent organs or from cystic complications. Poorly tolerated cysts, especially those located under the costal margin or in the epigastric region, can cause localized pain and significantly impact quality of life [31, 36].

Beyond symptoms from mass effect, non-cystic hepatic parenchyma can be impaired due to hepatic venous outflow obstruction, leading to portal hypertension and associated complications such as ascites, variceal hemorrhage, or hypersplenism. A French retrospective study of hepatic resection specimens or explants found sinusoidal dilation in 92% of cases within the “healthy” hepatic parenchyma, suggesting obstruction of venous return [38]. Notably, hepatic cysts do not cause liver failure. However, liver function abnormalities are frequently observed, primarily cholestatic in nature, due to bile duct compression, activation of biliary cells, or microcirculatory abnormalities.

Acute hemorrhagic or infectious events can complicate polycystic liver disease. Intracystic hemorrhage is common and presents with pain, the duration of which correlates with the size of the hemorrhagic cysts. Recurrence of hemorrhage can lead to iron deficiency anemia [39].

### 5.3 Imaging

The diagnosis of PLD is typically made when the number of hepatic cysts exceeds 20, or more than 4 in a patient with a family history of isolated PLD [40]. Distinguishing the type of PLD can be challenging, as patients with isolated PLD may have renal cysts, and those with ADPKD or ARPKD may have hepatic cysts as the primary clinical feature of their polycystic disease.

**Fig. (6):**
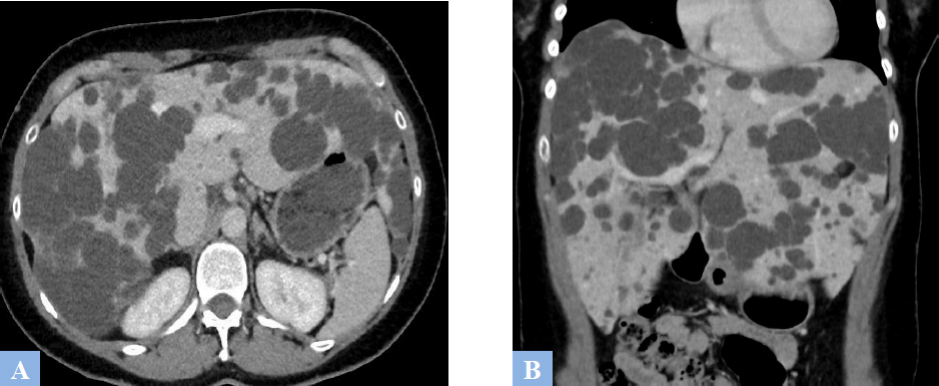
Uncomplicated hepatic polycystic disease. **A.** CT image in the axial plane at the portal phase of enhancement. Multiple well-defined hypodense hepatic cysts involving all hepatic segments. **B.** Same CT scan, coronal reconstruction. Liver enlargement due to the multiple biliary cysts.

**Fig. (7):**
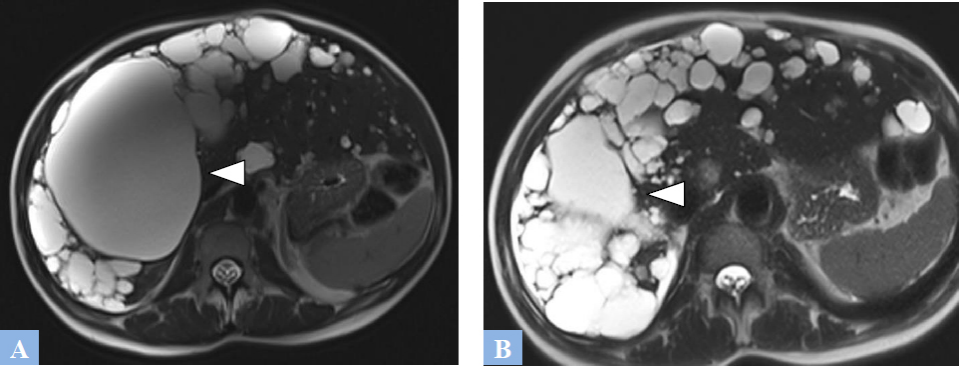
LD in a symptomatic 44-year-old female patient. **A.** T2-weighted MR image in the axial plane. Predominant cyst in the right liver (arrowhead). **B.** T2-weighted MR image in the axial plane, follow-up 3 months after percutaneous alcohol ablation of the predominant cyst (arrowhead).

On imaging, the cysts vary in size, often being supracentimetric, and exhibit the typical features of simple cysts: well-defined, thin walls, absence of septations or mural nodules, and posterior acoustic enhancement on ultrasound. In cases with numerous cysts, the liver's architecture is significantly altered, obscuring usual anatomical and vascular landmarks. Intracystic changes, such as hemorrhages and infections, are common. Cross-sectional imaging shows that cyst walls do not enhance after contrast injection, and calcifications are rare (Fig. **6**). MRI is particularly valuable for detecting complications (Fig. **7**), as it can identify hyperintense signals on T1-weighted images, indicative of intracystic hemorrhage [3].

Locating infected cysts can be difficult with conventional imaging due to frequent cystic changes from previous hemorrhagic episodes. In such cases, PET scans can be instrumental in pinpointing the infected cyst(s) or ruling out cystic infection by identifying an alternative extrahepatic infectious source [35].

Imaging also facilitates the counting of cysts and assessing their size, as well as determining the volume of parenchyma spared by the cysts. These parameters are integral to classifications such as those by Gigot and Schnelldorfer.

### 5.4 Treatment

**Fig. (8):**
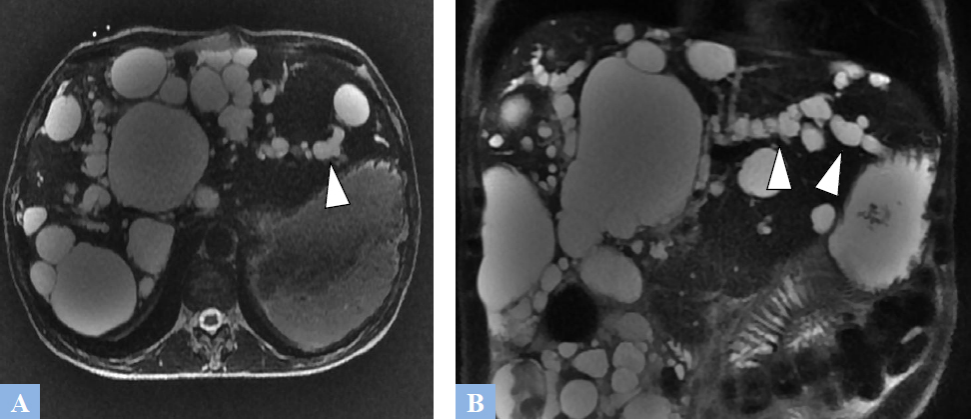
PLD in a 54-year-old patient presenting with non-febrile jaundice, leading to the indication for liver transplantation. **A.** T2-weighted fat-suppressed MR image in the axial plane showing hepatic polycystic disease with multiple peri-hilar cysts compressing the hepatic hilum, associated with upstream biliary duct dilation (arrowhead). **B.** T2-weighted fat-suppressed MR image in the coronal plane. Dilation of the intrahepatic bile ducts of the left lobe (arrowhead).

Aspiration and sclerotherapy are effective for managing symptomatic cysts larger than 4 cm, though recurrence rates necessitate repeated interventions (Fig. **8**). Arterial embolization has shown promise in reducing cyst volume in some studies [41, 42], but inconsistent results and risks, such as liver failure, prevent its routine use.

Fenestration is used to manage multiple cysts, often performed laparoscopically, though it carries risks of recurrence and complications such as ascites and hemorrhage [43]. Hepatic resection, indicated for severe cases with unaffected liver segments, offers symptom relief but is associated with significant morbidity and postoperative challenges [44].

Currently, the only curative option, liver transplantation is reserved for patients with severe symptoms, complications, or malnutrition. It provides excellent long-term outcomes, with no recurrence of PLD in the graft [45-47]. In combined polycystic liver and kidney disease, dual transplantation offers additional benefits, including a reduced risk of rejection [48].

## 6 MUCINOUS CYSTIC NEOPLASM

Mucinous Cystic Neoplasms of the Liver (MCN-L) are rare lesions of the liver and bile ducts, previously referred to as cystadenomas and cystadenocarcinomas. They were reclassified in the 4th and 5th editions of the WHO classification of tumors of the digestive system in 2010 into two entities: 1) MCN-L, characterized by the presence of ovarian-type stroma and no communication with the biliary tree, and 2) Intraductal Papillary Mucinous Neoplasms (IPMNs), which communicate with the bile ducts. This reclassification necessitates ovarian-type stroma for diagnosing MCN-L, leading to the reclassification of some cystic tumors without such stroma, especially in men, as intraductal papillary neoplasms [49].

### 6.1 Epidemiology, Origin, and Clinical Presentation

MCN-L are rare, representing less than 5% of intrahepatic cystic lesions, less than 5% of biliary tumors, and less than 0.5% of malignant liver tumors [8], with a prevalence estimated to be between 1 in 10,000 and 1 in 100,000. The presence of ovarian stroma in MCN-L suggests an origin from ectopic ovarian mesenchymal stroma that migrates to the liver or pancreas during embryonic development. It is hypothesized that bulging cells from the peritoneal surface epithelium of embryonic gonads can detach and attach to nearby organs, such as the liver, during early development [50]. Alternatively, MCN-L may result from hypersensitivity of fetal pancreatic and liver epithelium to female sex steroids, leading to proliferation.

MCN-L typically occurs in middle-aged Caucasian women (sex ratio > 9/1) and is usually not associated with invasive carcinoma. However, the risk of invasive carcinoma is believed to be higher in men. These lesions may present with pain, a mass effect (often due to gastric compression), or bile duct obstruction, but they can also be asymptomatic and discovered incidentally. The presence of ovarian-type stroma explains the higher prevalence of MCN-L in women; however, why some men develop MCN-L remains unclear. A similarity between ovarian-type stroma and stromal cells in the embryonic hepatobiliary tract is hypothesized [49].

### 6.2 Imaging

MCN-L are generally large and appear hypoechoic on conventional ultrasound, often with irregular thickened walls and internal echoes. They are typically described as multilocular masses with mural nodules and septa; however, a unilocular appearance may also be observed [6]. On CEUS, septal enhancement is noted during the arterial phase but disappears in the portal and late phases [8, 51]. Cystadenocarcinomas share similar features, with consistent septa and mural nodules that enhance in the arterial phase, sometimes exceeding 1 cm in thickness [52]. Differentiating benign MCN-L from cystadenocarcinomas can be challenging due to their similar enhancement characteristics. The absence of mural nodules and a unilocular appearance are key features that favor benign MCNs.

**Fig. (9):**
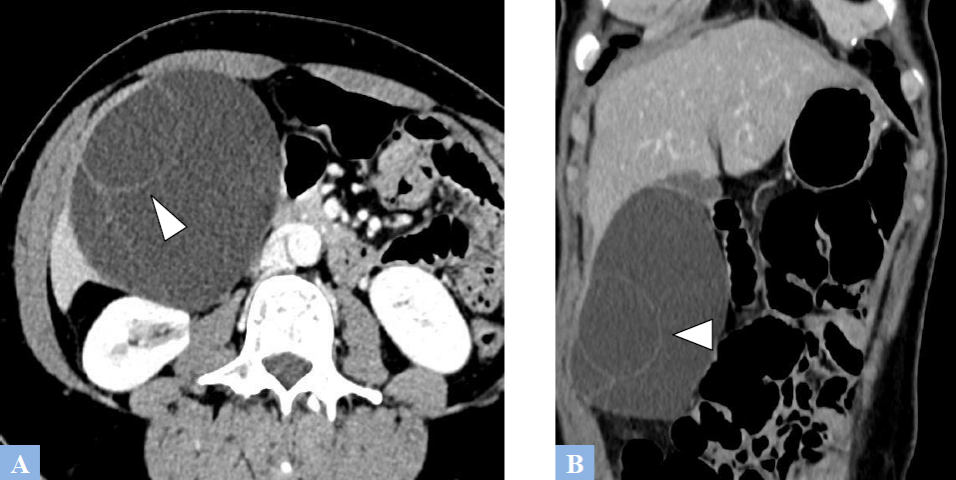
Low-grade mucinous cystic neoplasm (formerly mucinous cystadenoma) in a 37-year-old female patient presenting with abdominal heaviness. **A.** CT image in the axial plane at portal phase of enhancement. Rounded, exophytic cystic lesion in segment V, well-defined, containing thin septations enhanced after contrast injection (arrowhead). **B.** Same study, coronal reconstruction showing intracystic septations enhanced after contrast injection (arrowhead).

**Fig. (10):**
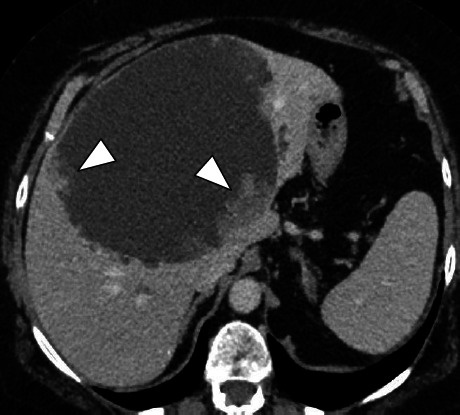
CT image in the axial plane at portal phase of enhancement. High-grade mucinous cystic tumor (formerly mucinous cystadenocarcinoma). Large cystic lesion in segment IV containing irregular, enhanced intracystic vegetations after contrast injection (arrowhead).

**Fig. (11):**
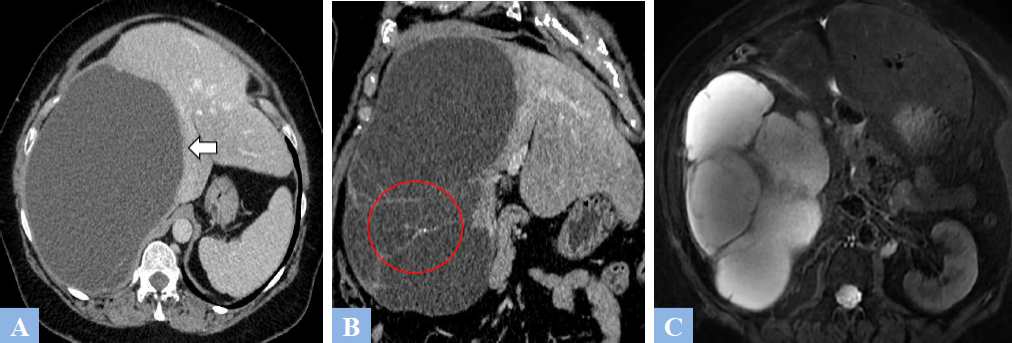
Mucinous cystic neoplasm in a 57-year-old female patient. CT and MRI features. **A.** CT image in the axial plane at portal phase of enhancement. Large cystic lesion in the right liver (arrow) displacing the remaining liver to the left. **B.** Same study, coronal reconstructions showing enhanced intracystic septations after contrast injection at the lower part of the lesion, associated with fine septal calcifications (red circle). **C.** T2-weighted fat-suppressed MR image in the axial plane. Septations appearing as T2 hypointense (red circle) within a T2 hyperintense fluid-filled cystic lesion.

On CT scans, MCN-L often exhibit thickened or irregular cyst walls and septa (Figs. **9**-**11**), with the latter more frequently observed compared to ultrasound, as shown in a single-center American study of 22 MCN-L over 17 years [53]. Occasionally, pericystic dilation of intrahepatic bile ducts can be seen.

MRI reveals well-defined lesions that are hypointense on T1 and hyperintense on T2, typical of fluid signals, without enhancement following gadolinium administration. MRI effectively delineates the internal architecture, including nodules and septa (Fig. **11**). A spontaneous hyperintense T1 signal, which may suggest hemorrhagic content, is sometimes considered indicative of malignancy [3]. MCN-L does not communicate with the bile ducts, and MRI does not specifically highlight the ovarian-type stroma.

### 6.3 Differential Diagnosis

Differentiating MCN-L from hepatic abscesses and hydatid cysts [54] relies on a septic context, endemic exposure, and hydatid serology for confirmation.

**Fig. (12):**
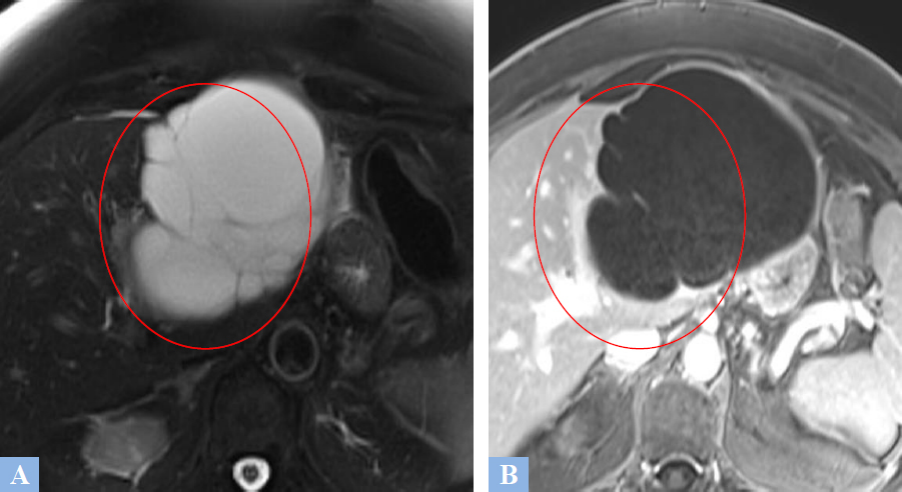
Multiloculated biliary cyst mistakenly diagnosed as a mucinous cystadenoma in a 67-year-old female patient. **A.** T2-weighted fat-suppressed MR image in the axial plane. Lobulated cystic lesion containing intracystic septations appearing as T2 hypointense. **B.** T1-weighted fat-suppressed MR image in the axial plane after gadolinium administration at the portal phase of enhancement showing mild enhancement of the intracystic septations (red circle).

Ultimately, atypical biliary cysts pose the most significant challenge due to their high frequency and the radically different management required for biliary cysts and MCN-L [55]. In this context, certain highly suggestive signs of MCN-L, visible on CT and MRI, are very useful to know in order to promptly indicate surgical resection: bile duct dilation upstream of the tumor (more visible on MRI than on CT), contrast enhancement of the walls, and the presence of mural nodules (fleshy projections). These signs are detectable with good inter-reader agreement. They are sufficiently predictive that the combined presence of 2 or 3 of them in a single lesion has a specificity of more than 94% for the diagnosis of MCN-L [49]. Conversely, the presence of at least 3 additional cystic lesions with a typical biliary cyst appearance is a strong argument for diagnosing an atypical biliary cyst rather than an MCN-L. Another particularly discriminating sign is the presence or absence of an indentation at the base of the septa, associated with an external convexity of the cyst wall on either side of each septum (Fig. **12**). In Kovacs' study [56], this sign alone was able to distinguish between adjoining biliary cysts and a mucinous cystic tumor, with a specificity of 86% for diagnosing MCN-L (in the absence of indentation) and a specificity of 91% for diagnosing biliary cysts if indentations are visible. These results were confirmed in a second study [57], where the specificity of the presence of indentations for diagnosing simple biliary cysts reached 100%, while the absence of indentations had only a 56% specificity for diagnosing MCN-L. In this challenging diagnostic context, recent advances in imaging technologies, particularly dual-energy and photon-counting CT [58-60], which enable enhanced visualization of subtle contrast uptake in septa and mural nodules, may significantly improve lesion characterization. Dedicated studies are warranted to further explore these promising modalities, potentially refining diagnostic criteria and patient management strategies.

### 6.4 Treatment

The treatment for MCN-L in the absence of evidence-based recommendations is surgical resection, typically lobectomy, which offers an excellent prognosis with nearly 100% 5-year survival [61]. Fenestration and percutaneous treatments are associated with high recurrence rates and should be avoided whenever possible. In cases of diagnostic uncertainty, intraoperative frozen section examination can guide the appropriate surgical approach [62]. There are no targeted therapies for cystadenocarcinomas associated with MCN-L, and management remains challenging with limited success reported for radiotherapy and chemotherapy [63].

## 7 INTRADUCTAL PAPILLARY MUCINOUS NEOPLASMS

Intraductal Papillary Mucinous Neoplasms (IPMNs), previously referred to as “biliary papillomatosis,” are characterized by their connection with the bile ducts and the subsequent dilation of downstream bile ducts due to the production of intraluminal mucin. IPMNs can arise anywhere along the biliary tree and exhibit varying degrees of malignant potential. They are classified into five stages: 1) IPMNs with low-grade or intermediate-grade intraepithelial dysplasia, 2) IPMNs with high-grade dysplasia, 3) IPMNs with carcinoma in situ, 4) IPMNs with microscopic invasion, and 5) IPMNs associated with invasive carcinoma [31, 49].

The precise etiology of these tumors remains incompletely understood; however, chronic inflammation of the bile ducts is believed to play a critical role in the pathogenesis and oncogenesis of IPMNs. This inflammation may result from factors such as biliary lithiasis, infection with *Clonorchis sinensis* (particularly prevalent in Asia), and reflux of pancreatic fluid into the bile ducts, often associated with an elongated common bile-pancreatic duct [49].

### 7.1 Epidemiology and Clinical Presentation

IPMNs are exceedingly rare, with fewer than 300 cases reported in the literature. They predominantly affect middle-aged individuals between 40 and 70 years old, with a slight male predominance. The highest incidence rates are observed in East Asian countries, including Japan, Korea, and Taiwan. While IPMNs can be asymptomatic, they frequently present with clinical symptoms such as right upper quadrant pain, recurrent episodes of cholangitis, or intermittent obstructive jaundice. Biliary obstruction may result from factors such as common bile duct stones, detachment of tumor fragments, or excessive mucin secretion.

### 7.2 Imaging

In imaging, Intraductal Papillary Mucinous Neoplasms (IPMNs) are classified into four types [64]:

(1) Type 1: Intraductal mass associated with upstream ductal dilatation.

(2) Type 2: Marked bile duct dilatation without a visible mass.

(3) Type 3: Visible mass with both upstream and downstream bile duct dilatation.

(4) Type 4: Cystic dilatation of the bile duct with a papillary mass (55).

Ultrasound can detect bile duct dilation and its potential consequences, such as the presence of stones; however, its sensitivity for detecting intraductal masses is low. Contrast-enhanced CT typically reveals intraductal masses that enhance during the arterial phase and wash out during the portal phase. This characteristic helps distinguish IPMNs from cholangiocarcinomas, which typically have a fibrous component that results in persistent and increasing enhancement in the late phases [49]. Despite this, the sensitivity of CT for detecting IPMNs is around 50%, which remains inadequate.

On MRI, IPMNs forming an intraductal mass exhibit much lower signal intensity compared to bile on T2-weighted images, facilitating their detection. Diffusion-weighted MRI may improve the identification of invasive carcinoma. However, Type 2 IPMNs can be particularly challenging to detect on CT and MRI, as the mucinous tumor is often iso-dense on CT and iso-intense on MRI relative to bile. For these cases, MR cholangiography is required to identify the “thread sign,” a poorly sensitive but highly specific indicator of IPMN (specificity > 99%), characterized by hypointense linear or curvilinear intraductal streaks within dilated bile ducts [65].

Type 4 IPMNs may be misinterpreted as simple bile duct cysts, especially when the papillary mass is not visible or is overlooked within a large cystic volume. Detecting communication between the cystic lesion and the bile duct on MR cholangiography is crucial for confirming the diagnosis. The use of hepatobiliary contrast agents (*e.g.*, Multihance®) can further aid in identifying this communication [49].

Given the limitations of conventional imaging for IPMN detection, innovative imaging technologies such as photon-counting CT and advanced MRI sequences (*e.g.*, high-resolution diffusion imaging or ultra-short TE sequences) represent promising avenues for improving lesion detection and characterizing subtle intraductal features.

There is limited data on the role of FDG-PET in the management of IPMNs, but it may be useful in detecting high-grade dysplasia or invasive carcinoma. ERCP can identify tumors as irregular filling defects within the bile duct walls and observe the flow of mucinous fluid through the papilla. Cholangioscopy allows for direct visualization of the solid components of an IPMN, assessment of its extent within the bile ducts, and the performance of brushings and biopsies to confirm the diagnosis [66, 67].

### 7.3 Treatment

Surgical resection is the main treatment for IPMNs, with liver transplantation considered in some cases [68]. This approach carries risks of recurrence and requires lifelong monitoring. Alternatives include bile duct drainage and local ablation therapies when surgery isn’t an option. IPMNs have a better prognosis than cholangiocarcinomas; however, factors such as positive margins and invasive components worsen outcomes [49].

## 8 CILIATED HEPATIC FOREGUT CYST

The ciliated hepatic cyst, also known as a congenital ciliated hepatic cyst of the foregut (CHFC), is an extremely rare lesion typically located in the subcapsular region of segment IV. Its wall is lined with pseudostratified ciliated columnar epithelium overlying connective tissue, smooth muscle fibers, and a peripheral fibrous capsule [69]. There is no bile duct present. It is thought that these cysts arise from an early and abnormal budding of the foregut, which is the precursor of the liver.

**Fig. (13):**
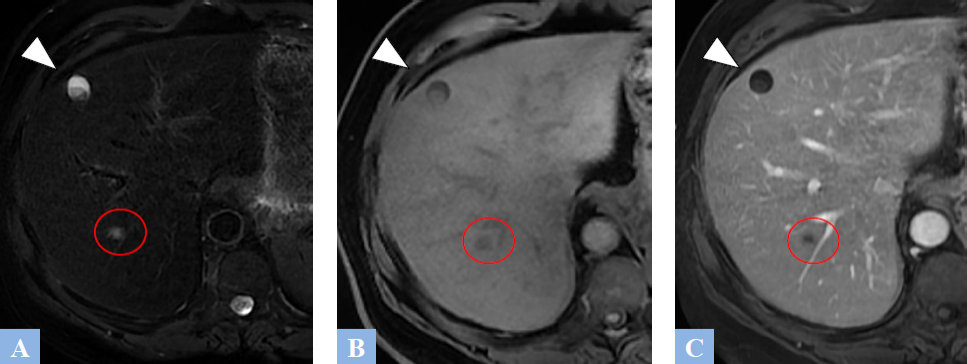
Ciliated hepatic foregut cyst in a 60-year-old male patient undergoing treatment for a metastatic colorectal cancer. **A.** T2-weighted fat-suppressed MR image in the axial plane showing a well-defined cystic mass in segment IV, which is markedly hyperintense on T2 (arrowhead). A more posterior metastatic lesion is noted, with poorly defined margins, exhibiting an intermediate T2 signal (indicated by the red circle). **B.** T1-weighted fat-suppressed MR image in the axial plane. The cystic lesion in segment IV appears hypointense, with hyperintense dependent sediment (arrowhead). The metastasis also exhibits T1 hypointensity, though it is less pronounced (red circle). **C.** T1-weighted fat-suppressed MR image in the axial plane after gadolinium administration at the portal phase of enhancement showing no cystic enhancement (arrowhead). The secondary lesion, in turn, shows poorly defined peripheral enhancement (red circle).

On imaging, the lesion measures less than 4 cm, and its typical location should immediately suggest the diagnosis. There may be posterior enhancement on ultrasound. On CT, the cyst's density may appear slightly lower than that of a simple bile duct cyst. On MRI, the internal signal is slightly more hypointense on T2 and hyperintense on T1 compared to the signal of a simple cyst. Fluid-fluid levels are often observed, and there is no enhancement after intravenous contrast injection (Fig. **13**).

CHFC can be symptomatic due to its subcapsular location [70]. They are described as benign lesions, but there have been reports of a potential for malignant transformation into squamous cell carcinoma [71], which justifies prophylactic surgical resection once the diagnosis is confirmed [3, 49].

## 9 PERIBILIARY CYSTS

Peribiliary cysts are macroscopically visible cystic dilations of the peribiliary glands. They are located in the hepatic hilum and can measure up to 1 cm in diameter. They have been described in association with congenital polycystic liver diseases and cirrhosis [72]. A considerable number of patients with cirrhosis and chronic alcoholic pancreatitis have peribiliary cysts, and it has been suggested that alcoholic liver disease, as well as its association with chronic pancreatitis, are conditions that promote the formation of peribiliary cysts, with their presence being closely correlated with the extent of pancreatic fibrosis. The fibrosis of the peribiliary glands is indeed the key element in the formation of peribiliary cysts. It may be secondary to the activation of hepatic stellate cells but is primarily caused or exacerbated by alcohol-induced adenitis, which leads to scar fibrosis of the peribiliary glands [73].

**Fig. (14):**
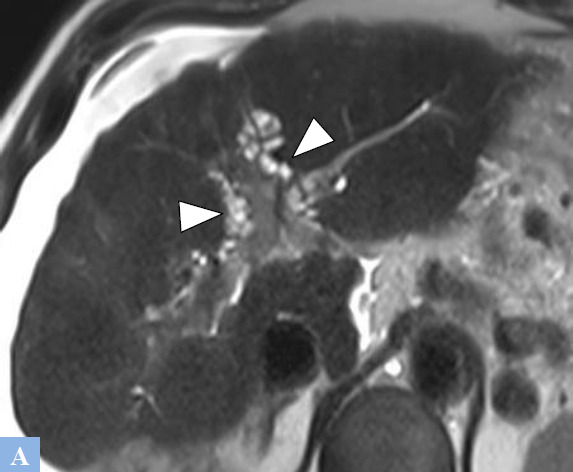
Peribiliary cysts in a patient with a history of alcohol-related cirrhosis. T2-weighted fat-suppressed MR image in the axial plane. Multiple centimeter-sized cystic lesions adjacent to the proximal bile ducts (arrowheads).

On imaging, peribiliary cysts can appear as discrete cystic clusters at the level of the portal bifurcation, as a chain of cysts mimicking abnormal bile ducts, or as a tubular structure parallel to the portal structures near the hepatic hilum. Peribiliary cysts are anechoic and show no detectable flow on Doppler examination. On CT, peribiliary cysts are hypodense with no contrast enhancement. On MRI (Fig. **14**), peribiliary cysts appear hyperintense on T2-weighted images and hypointense on T1-weighted images, with no enhancement after gadolinium administration. They often present with characteristic features of chronic pancreatitis: pancreatic atrophy, parenchymal calcifications, and irregular dilation of the pancreatic ducts. It is essential to distinguish between peribiliary cysts and genuine dilated bile ducts, particularly in the context of cirrhosis, to avoid unnecessary interventions [74]. Indeed, peribiliary cysts do not complicate and do not undergo malignant transformation.

## 10 OCCASIONALLY CYSTIC-APPEARING LESIONS

Many lesions that are not classified as true cysts can occasionally present as hepatic cysts. In most cases, the clinical context allows for easy diagnosis [3, 75].

### 10.1 Benign Tumors

#### 10.1.1 Cystic Hemangioma of the Liver

Some hemangiomas can rarely present in a cystic form and may be challenging to characterize on imaging [76] when the progressive enhancement after contrast injection, typical of hemangiomas, is poorly visualized.

#### 10.1.2 Mesenchymal Hamartoma

Mesenchymal hamartomas are the second most common benign hepatic tumors. They typically affect children, usually before the age of three [77]. The internal architecture varies: solid, cystic, or mixed. In their cystic presentation, mesenchymal hamartomas appear as large, multilocular lesions on imaging. Age helps to differentiate this lesion from cystadenomas, which typically affect middle-aged women.

### 10.2 Traumatic or Surgical Condition

#### 10.2.1 Hematomas

Hematomas are generally easy to differentiate from other cystic lesions due to the presence of a history, sometimes dated, of trauma or surgical or percutaneous intervention. They can be located subcapsularly or within the hepatic parenchyma. Depending on their stage of evolution, they may appear hyperdense on CT scans and hyperintense on T1-weighted MRI, indicating the presence of blood; however, these characteristics can fade over time, and the lesion may eventually present as heterogeneous or even as a hypodense cyst on CT scans.

#### 10.2.2 Biloma

A biloma is an intra- or peri-hepatic collection resulting from spontaneous or iatrogenic trauma to the bile ducts. It is most commonly observed following biliary surgery and appears as a well-defined anechoic collection near the hepatic pedicle or the gallbladder. There is no enhancement on CT [3]. In cases of diagnostic uncertainty, a puncture revealing a bilirubin-rich fluid has the additional benefit of ruling out surinfection and preventing choloperitoneum.

### 10.3 Lesions Specific to Women of Childbearing Age

#### 10.3.1 Liver Endometriosis

An endometriotic cyst is a rare hepatic parenchymal or subcapsular lesion that may appear complex on imaging, featuring a nodular and septal clot and/or blood degradation products. The presence of periodic epigastric or right upper quadrant pain, along with the coexistence of another site of endometriosis, helps in making the diagnosis [78].

#### 10.3.2 Intrahepatic Ectopic Pregnancy

In a young woman presenting with amenorrhea (though it may be inconsistent) and right upper quadrant pain, a cystic lesion typically affecting the right hepatic lobe may correspond to the gestational sac of an exceptionally rare intrahepatic ectopic pregnancy. The fetus, when of sufficient size, can be clearly visible on a scan. It is often still alive at the time of diagnosis. Surgical intervention is necessary to prevent a hemorrhagic rupture of the liver capsule when the lesion is large, with or without prior methotrexate injection to terminate the pregnancy [79].

### 10.4 Neoplastic Setting

Several primary or secondary neoplastic lesions can appear cystic or pseudocystic. This appearance may result from cystic differentiation or intratumoral necrosis [3].

#### 10.4.1 Primary Lesion

Among primary tumors, hepatocellular carcinoma [80] and cholangiocarcinoma can exceptionally present as cystic lesions [81], as can some primary sarcomas of the liver [3]. The cystic appearance can occur after a completely effective treatment (*e.g.*, sequestration post-thermoablation of a hepatocellular carcinoma) or a partially effective one, such as chemoembolization, targeted therapy, or conventional chemotherapy.

#### 10.4.2 Cystic Metastasis

**Fig. (15):**
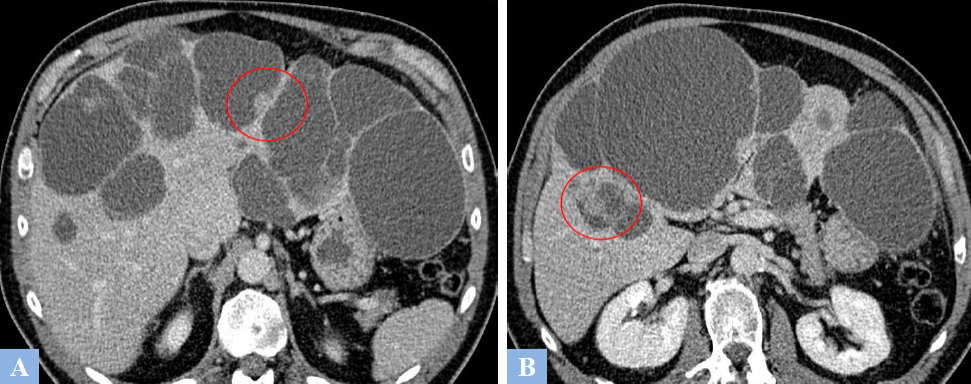
Cystic metastases from a rectal neuroendocrine tumor discovered in a 54-year-old patient presenting with right hypochondrium pain. **A.** CT image in the axial plane at the portal phase of enhancement. Multiple hepatic cystic lesions with intracystic vegetations (red circle) are consistent with secondary lesions. **B.** Same study, lower slices. Multiple hepatic cystic secondary lesions with intracystic vegetations (red circle).

**Fig. (16):**
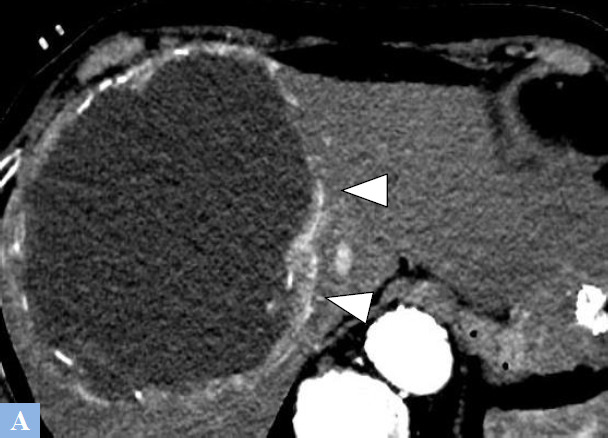
Cystic metastasis from a gastric GIST. CT image in the axial plane at portal phase of enhancement showing mixed lesion, both solid and cystic, in the right liver with a hypervascular wall (arrowheads).

Cystic liver metastases are common and can be associated with several primary cancers, including sarcomas (such as myxoid liposarcoma and embryonal sarcoma affecting children), neuroendocrine tumors [82, 83] (Fig. **15**), gastrointestinal stromal tumors (Fig. **16**), mucinous gastrointestinal and ovarian tumors, and, of course, metastases from pancreatic cystadenocarcinoma.

Approximately 50% of cystic liver metastases originate from colorectal cancer [2]. These cystic metastases are often multiple. The most useful radiological sign for diagnosis is the presence of wall enhancement after contrast injection on CT, MRI, or contrast-enhanced ultrasound, which immediately suggests a tumor nature of the lesions [3, 49]. When the neoplastic context is unknown and the primary tumor is not identified, histological examination is essential and justifies a percutaneous biopsy [84].

## CONCLUSION

In conclusion, non-infectious hepatic cystic lesions encompass a wide range of lesions, from benign to potentially life-threatening. Accurate diagnosis should be performed using imaging techniques, including ultrasound, CT, MRI, and CEUS, when necessary. Emerging imaging techniques, such as dual-energy CT, photon-counting CT, and advanced MRI sequences, hold promise for detecting subtle enhancement patterns that are not visible with conventional imaging. Additionally, artificial intelligence (machine learning and deep learning) may improve lesion characterization, predict malignancy, and identify complications or atypical features. Nevertheless, despite these technological advances, detailed knowledge of clinical context and the natural history of hepatic cystic lesions will always be essential for radiologists, as it provides critical guidance for image interpretation and understanding of lesion-specific imaging characteristics.

## AUTHORS’ CONTRIBUTIONS

The authors confirm their contributions to the paper as follows: P.C.: Study conception and design; D.W.: Conceptualization; E.D.: Visualization; A.B.C.: Providing figures; A.C., V.D.M.: Drafting the manuscript. All authors reviewed the results and approved the final version of the manuscript.

## Figures and Tables

**Fig. (4):**
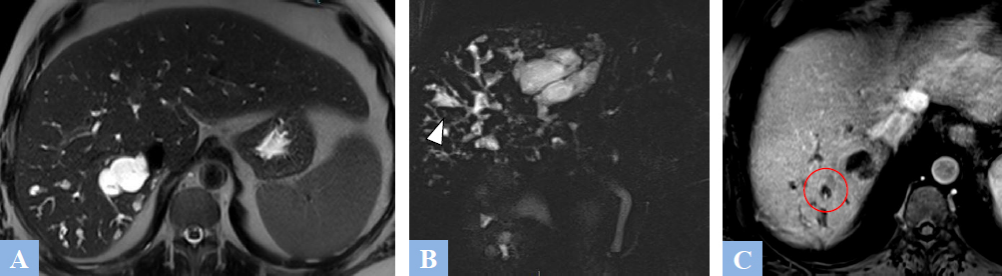
Examples of biliary hamartomatosis. **A.** T2-weighted MR image in the axial plane. Numerous subcentimeter cystic lesions with T2 hyperintensity across all hepatic segments. **B.** 2D radial MR cholangiography showing countless millimetric hepatic cystic lesions creating a “starry sky” appearance. **C.** 2D radial MR cholangiography. Multiple larger hepatic cystic lesions are consistent with biliary hamartomas.

**Table 1:** Major pitfalls encountered in the morphological assessment of biliary cysts.

**Trap Images**	**Possible Explanations**	**Elements Restoring the Diagnosis**
Solid-appearing image inside the cyst or on its wall.	1. Intracystic hemorrhage 2. Cyst infection 3. Wall rupture	1. Dependent sediment (ultrasound) hyperintense on T1 (MRI). 2. Wall thickening / cloudy fluid on puncture. 3. Perihepatic effusion; serpiginous intracystic element (CT) mobile (ultrasound) corresponding to the ruptured wall.
Presence of septa (suggestive of cystadenoma), altered cyst appearance	1. Several adjacent cysts 2. Hemorrhagic alterations 3. Post-sclerosis alterations	1. Absence of wall thickening, vegetation, contrast enhancement of walls or septa, perilesional biliary dilatation, and PET scan activity. 2. Presence of indentation of the walls in line with the septa. 3. Same + Hyper T1 signal of the pseudo-septa. 4. Medical history.
Parietal calcification	1. Post-hemorrhagic alteration?	1. Absence of wall thickening, pericyst, proliferative membrane, or daughter vesicles suggestive of hydatid cyst. 2. Absence of fleshy bud or enhancement after injection, suggestive of a neoplastic lesion. 3. Absence of septa suggestive of cystadenoma.

**Table 2:** Summary of main imaging features of non-infectious liver cystic lesion.

**Lesion**	**Radiologic Semiology**
Biliary cyst	Rounded, homogeneous, well-defined borders, no visible wall Anechoic (US), hypodense < 20 UH (CT), strongly hyperintense on T2 (MRI)
Hamartoma	Small cystic lesions < 1cm, well-defined, uniform in size, diffuse distribution No communication with the biliary ducts Comet tail artifact (US), hypodense without enhancement (CT), hyperintense on T2, “starry sky” or “snowstorm” (MRI)
Caroli disease	Communication between cystic lesions and normal bile ducts, Dot sign (CT). MR cholangiography: multiple cystic ectasies with fusiform dilatations / isolated fusiform dilatations with stones Association with congenital hepatic fibrosis: dysmorphic, signs of portal hypertension
Polycystic liver disease	Numerous hepatic cysts (> 20) or fewer if family history Well-defined, thin walls, no septations or mural nodules, anechoic (US), hypodense < 20 UH (CT), strongly hyperintense on T2 (MRI) Value of MRI for detecting complications, hyperintense signals on T1: intracystic hemorrhage
Mucinous cystic neoplasm	Well-defined lesions, thickened walls and septa, mural nodules, and hemorrhagic content are possible. Septa and mural nodule enhancing (CEUS, CT, or MRI) Difficulties in differentiating benign from malignant lesions
Intraductal papillary mucinous neoplasm	Intraductal mass with bile duct dilatation Inadequate sensitivity of CT and US in detection MRI: intraductal mass with lower signal intensity compared to bile on T2, MR cholangiography: “thread sign”
Ciliated hepatic foregut cyst	Well-defined borders, rounded, typical location, less than 4 cm, no visible wall Density slightly lower on CT, signal slightly more hypointense on T2, and hyperintense on T1
Peribiliary cyst	Well-defined, rounded, no visible wall, cystic clusters at typical locations mimicking abnormal bile ducts Association with cirrhosis, chronic alcoholic pancreatitis, and polycystic liver diseases Anechoic (US), hypodense with no enhancement (CT), strongly hyperintense on T2 with no enhancement (MRI)

## LIST OF ABBREVIATIONS

ADPKD: Autosomal Dominant Polycystic Kidney Disease
ARPKD: Autosomal Recessive Polycystic Kidney Disease
CEA: carcinoembryonic antigen
CEUS: Contrast-enhanced ultrasound
CHFC: Ciliated Hepatic Foregut Cyst
CT: Computed Tomography
MCN-L: Mucinous Cystic Neoplasm of the Liver
IPMN: Intraductal Papillary Mucinous Neoplasm
PET: Positron Emission Tomography
PLD: Polycystic Liver Disease
ERCP: Endoscopic retrograde cholangiopancreatography
MRI: Magnetic Resonance Imaging
TIPS: Transjugular Intrahepatic Portosystemic Shunt
US: Ultrasound
